# How Blue is the Sky?

**DOI:** 10.1523/ENEURO.0130-21.2021

**Published:** 2021-04-27

**Authors:** Yves Frégnac

**Affiliations:** UNIC-NeuroPSI, Institut des Neurosciences Paris-Saclay, Centre National de la Recherche Scientifique, Gif-sur-Yvette 91190, France

**Keywords:** big data, brain modeling, high-performance computing, IT, neuromorphic, neuroscience

## Abstract

The recent trend toward an industrialization of brain exploration and the technological prowess of artificial intelligence algorithms and high-performance computing has caught the imagination of the public. These impressive advances are fueling an uncontrolled societal hype, the more amplified, the more “Blue Sky” the claim is. Will we ever be able to simulate a brain *in silico*? Will “it” (the digital avatar) be conscious? The Blue Brain Project (BBP) and the European flagship the Human Brain Project (HBP) have surfed on this wave for the past 10 years. Their already significant lifetimes now offer new case studies for neuroscience sociology and epistemology, as the projects mature. Their distinctive “Blue Sky” flavor has been a key feature in securing unprecedented funding (more than one billion Euros) mostly through supranational institutions. The longitudinal analysis of these ventures provides clues to how the neuromyth they propagate sells science, in a scientific world based on an economy of promises.

## Significance Statement

This epistemological review examines how two recent global initiatives which focus on the possibility of simulating the Human Brain *in silico*, the Blue Brain Project (BBP) and the Human Brain Project (HBP), have recently caught the imagination of the public. We explore how the scientific roadmaps have been shaped to motivate an interdisciplinary paradigm shift in neuroscience research under a blue-skies research banner, allied with evolving advanced information and communication technology (ICT) tools, together having the potential to trigger a quantum leap in applied brain science. A longitudinal analysis reveals how the neuromyth of digitizing-the-mind has propagated to sell science, and how scientific goals have shifted and refocused over time.

## Mise en Abyme

Ten years or more have elapsed since the start of the Blue Brain Project (BBP; 2005–2013; for review, see Markram, 2006) and Henry Markram’s original claim, that he would build a digital virtual version of the Human Brain within the next 10 years. This call to action was aired during the famous 2009 TED-Global Talk, which has been seen now by >1.5 millions viewers. Its impact triggered a massive industrialization of Neuroscience data mining. In just a few years, there has been a global shift in experimental and theoretical paradigms in brain studies, which has spread all over the world, opening the era of Global Neuroscience (Markram, 2012; Alivisatos et al., 2013; Jorgenson et al., 2015; Grillner et al., 2016; for review, see Frégnac and Laurent, 2014; Frégnac, 2017). The Human Brain Project (HBP), initiated in 2013, now comes to maturity and the clock signaling the end line is ticking loudly. There have now been 10 years of intense work to produce the promised digital simulation of the human brain. Ten years to convince a skeptical neuroscience community that the dream will eventually come true. Ten years of hope for significant progress, facing the same endgame disillusion that followed the “winterfall” of artificial intelligence (Lighthill, 1973) when AI failed in its promise to emulate human intelligence.

A first epistemological account of the BBP, and its twin, the HBP, has been “mis en abyme” by documentary writer and film director Noah Hutton, in his series of yearly chronicles (“BlueBrain.com”; Hutton, 2012). His final synthesis (*In Silico*) is the starting point of my commentary (see below, The *In Silico* A Propos). This documentary constitutes a sociological narrative of a neuromyth, with all the necessary actors and artifacts: a charismatic leader (the storyteller), a layman (neuroscientist or not), and the walls of Plato’s cave (translation by Rouse, 1956). Visualized in the “blue room” at the Brain-and-Mind Institute (EPFL), multiscale atlases of the complex inner structure of the neocortex and movie clips of activity spread across distributed virtual neuronal assemblies are back-projected on the 2D-screen of a HD video-wall, to mesmerize the viewer. In the background, a collection of respected allies and competitors from large-scale global neuroscience initiatives and key partners [funding agencies, including the European Commission Future and Emerging Technologies (FET) program] are interviewed by Hutton, sometimes scratching their heads.

The storyteller pushes the theory that “the brain creates, builds, a version of the universe, and projects (it), like a bubble, all around us” (Markram, 2009). The “blue room” is a cave, now in the sense of graphic data engineering, where any visitor can gain access to the holy grail visions. What is mapped on the video screen is a dynamic cartography intended to reflect the canonical signature of circuits and mind processes in action in the brain. State-of-the-art immersive data visualization techniques operate the virtual reality platform: according to Henry Markram’s own poetic terminology, the fleeting “butterflies of soul” (De Felipe, 2017; Fan and Markram, 2019), the elusive “ghost-like structures” (Markram, 2009) and “sandcastles” (Reimann et al., 2017), that, in his outstanding experimental studies, he sometimes glimpsed during multiple simultaneous recordings *in vitro*, are now embodied *in silico*. To make the viewer’s immersion still more effective, the virtual simulations are animated in a slow-motion, waxing and waning in synchrony with the beat of the Johann Strauss’s Blue Danube. Blue room, blue sound, blue gene (IBM^TM^), blue brain…

This is where the story here meets the allegory of the Greek philosopher’s cave. In Plato’s tale, Socrates describes a group of people, chained to the wall of a cave, facing a blank wall all their lives. The prisoners watch shadows projected on the wall, the images of objects passing in front of a fire that is behind them, and give names (!) to these shadows. But Socrates, in his wisdom, tells us that while the shadows are deceivingly the prisoners' reality, they are not accurate representations of the real world. Indeed, what kind of in-depth knowledge can be gained by solely archiving of virtual ghosts and ephemeral constructs? Neuropsychologists are still struggling to understand the disconnection between the objective reality of the physical world, the individual variability of brain encoding and neural representations, and the subjectivity of conscious reports and perceptual illusions in humans (Ramachandran and Blakeslee, 1998). In the open-loop *in silico* brain, the functional validation of the simulations appears as an overwhelming task, since these are constrained largely by *in vitro* data (recorded in rat or mouse brain tissue slices at room temperature) in the absence of any link with cognitive behavior, percepts, or illusions.

## The Neuromyth

To explore the putative benefits of “comparative epistemology,” Jur Koksma has made a case study of the HBP 10-year storyboard and presents, in his seminal paper “Narrators of Neuromyth” (2020), a convincing neurocultural analysis based on the statements made by Henry Markram in both the public and scientific domains. The “Neuromyth” qualification is not to be seen forcibly as a negative statement, since it may reflect a positivist attitude toward science, grounding a change of paradigm (Giere, 2006). This name suits the present case well, because it involves an “origin story,” a claim to discover some ultimate truth about the physical nature of the Mind. It is used here to justify both a promise and a shift in scientific paradigms. For BBP and HBP, the original credo and the promise are that we will one day understand the brain, and that, in 10 years, we will be at least able to simulate it digitally. For Henry Markram, although we do not at present fully understand the brain, it is already time to start to build a digital emulation, based on what we already know from past animal experimentation. His act of faith is that knowledge should expand through virtual simulations in a kind of autocatalytic manner, as if they had the status of “true” experimental observations: by multiplying the number of realistic instantiations of possible connectomes, one could envision the reconstruction of a plausible full brain, realistically connected in the statistical sense, by applying a kind of “bootstrap” logic.

In fairness to the evolution of the initial projects (see Jirsa, 2021; Destexhe, 2021), it is worth acknowledging that the account of the “origin” story progressively changed from the BBP to the HBP projects, leaving the “blue sky” brain-and-mind issue on the side, to gradually take a form that would be more sellable to the information and communication technology (ICT) community. “Understanding the human brain is one of the greatest challenges facing 21st century science. If we can rise to the challenge, we can gain profound insights into what makes us human, develop new treatments for brain disease and build revolutionary new computing technologies. Today, for the first time, modern ICT has brought these goals within sight.” (Markram et al., 2011; Markram, 2013). The Flagship initiative was developed following a decision that Europe should reinforce its support for FET research under the ICT theme, to stimulate and explore new forms of multidisciplinary research collaboration going beyond existing organizational structures and models and reinforce its capability for permanent foresight of future research trends in ICT. Competition between potential flagship projects thus had a strong ICT dimension and this reformulation of the HBP goal also signaled a change in the selling strategy: the political move to strengthen the HBP ICT component was initiated during the final stage of the flagship competition, by Henry Markram together with Karlheinz Meier, a renowned Physicist and one of the founders of European neuromorphic research (for review, see Furber, 2016; Ravindran, 2019). This changing emphasis was later approved and put into gear by the mediator (Wolfgang Marquardt, also a Physicist) called to improve the scientific and technological roadmap of HBP, and its governance (for review, see Frégnac and Laurent, 2014). However, this abrupt change of course also explains the loss of trust on the part of the neuroscience community, which saw the takeover by ICT as dropping an essential part of the blue sky interest of the overall project (Waldrop, 2012; Frégnac and Laurent, 2014).

## The Microscope-Telescope Metaphor

From the start, it became obvious that the “Blue Sky” objective, the full simulation of the Human brain, was too high in the sky and would be difficult to achieve. Even within the HBP ramp-up phase, the project deliverables were quickly transformed to target building appropriate IT infrastructures and conceptualizing an optimal tool for brain-explorers: the microscope-telescope or “neuroscope” (Markram, 2013).

The microscope: as best exemplified by the Blue Brain image gallery, understanding the cortical design required reverse-engineered exploration of the Brain to visualize microscopic dimensions often thought unnecessary in neural network simulations (for the justification of mesoscopic modeling, see Destexhe, 2021), and to produce an exhaustive catalog of the neural rainforest, encapsulating myriads of branching dendritic trees, stereotypical somas, axons, and synaptic glomeruli.

The telescope: at a more mesoscopic level, big data and higher-dimensional parametric spaces were needed to extract significant replication of patterns across brains, both in the structural design (network connectome, local circuits, synaptic triads) and at the functional level (action potential firing patterns, neural assembly dynamics).

In terms of the history of science, this search strategy for detecting invariants, taking the form of prototypical morphologies at the cellular level, unexpectedly frequent functional connectivity patterns at the network level, or unusually high probability of specific correlated states at a more mesoscopic level, has a strong connotation: with, at the anatomic level, the random emergence of canonical neuromorphic anatomic forms of cells and circuits, as reconstructed by Ramón Y Cajal from Golgi-stained material, and, at the functional level, the spontaneous selection of active cortical states perceived as specific “planforms” during “entoptic vision,” as self-reported during drug-induced hallucination or migraines (Klüver, 1966; Tyler, 1978; for review, see Frégnac, 2003). In the words of Henry Markram during his TED talk, this search became daunting, metaphorical, and allegorical, boosting the excitement of the audience who would not have been surprised in the end to see an “electrical” homunculus sitting on some dendritic branch orchestrating the whole columnar assembly: “So, the way that we can look at that is to ignore the neurons, ignore the synapses, and look just at the raw electrical activity. Because that is what it’s creating. It’s creating electrical patterns. So when we did this, we indeed, for the first time, saw these ghost-like structures: electrical objects appearing within the neocortical column. And it’s these electrical objects that are holding all the information about whatever stimulated it. And then when we zoomed into this, it’s like a veritable universe.” However, despite the obvious poetic eloquence, this vision is nevertheless fed by a deep theoretical conviction: by archiving big data describing the structure and function of the brain, one might be able to identify a representational configuration space with a reduced number of dimensions (7–11), from which the activity graph characterizing the cortical state could be projected in meaningful snapshot blocks of variable and lower multidimensional geometry. “In a way, we are like flatlanders trying to understand 3D shapes” (Markram, 2020). Henry Markram and his colleagues have used algebraic topology (Munkres, 1984) to characterize a formal link between neural network structure and its emergent function (Reimann et al., 2017). Directed cliques describe the flow of information in local fully connected subnetworks while cavities, defined as collections of interconnected cliques with missing links, provide a global measure of information flow in the whole network. For Henry Markram, “when we see deeper, we see better the rules; the telescope (our visualization) will in itself catalyze discovery” (Markram, 2020). Some of us may be more skeptical (see Marder, 2021) and the sociologist Jur Kosksma sees there the pretense of a ‘magical’ hypothetical process where “models, when fed with rules, spit out new ones, increasing knowledge.”

Interestingly, from an historical perspective, we can see now how the publicized scientific target in BBP and HBP has evolved. There has been a progressive shift away from reading the mind and building a full digital Brain toward the constitution of Google-like databases, where modern neuroscientists and modelers can at will “zoom-in” (the “microscope”) or “zoom-out” (the “telescope”) through the BigData archives collapsed in a semantically annotated functional and structural multi-level atlas (Amunts, 2021). The knowledge quest is no longer to find the mechanisms of mind and emerging consciousness, but rather to build a multiscale description of the physical constitution of the Brain. For the “zoom-in” function, the present status of BBP/HBP research at the cellular and circuit level comes much in alignment with the long-established strategy of the Allen Institute. In parallel with the European scientists, this high-tech community engaged in a 10-year research effort, focusing on building brain atlases and big data viewing facilities such as the “mindscope” project (Koch and Reid, 2012). In this respect, Allen succeeded in providing the new Paxinos atlas, guided by a community of insiders (similar in size to BBP, but much smaller than HBP), which constitutes a state-of-the-art resource (review in Fairhall, 2021), mostly useful for mouse-ologists. At this stage of development, these global initiatives still avoid investment in more overt fundamental knowledge issues, than data taxonomy, data archiving and purely phenomenological modeling. Both initiatives have opened a Pandora’s box toward delving into ever more microscopic dimensions, and experimenters and theoreticians are now facing a sea of data and a formidable task of multidimensional integration.

## The Bottlenecks of the Imitation Game

This attractive way of representing morphologic regularities or activity pattern’s singularities has certainly its own virtues, by making neuroscientists aware that repeated correlated occurrences of certain patterns or events may carry more information than others. However, the expansion of the realm of “observable” brain patterns by simulation bootstrapping, makes the definition of a “null” hypothesis (to serve as a statistical reference) a difficult theoretical issue. It has also tortuous consequences: it takes us away from the classical data-driven approach to looking at the brain, using *in vivo* or *in vitro* animal experimentation. Blue Brain’s ambition (in its own words) is now to provide a third alternative way, *in silico*, “as if it was biologically real…”. Of course, the digital brain Ersatz, on which unlimited numbers of simulations can be run, will constitute an ever-reusable substrate for virtual experimentation and even personalized virtual medicine (Markram et al., 2011), which may open new perspectives, once we will get access to individual connectomes. However, the observation that the emergent virtual dynamics give rise to a complex array of network states comparable to that observed in real circuitry (Markram et al., 2015; Ramaswamy et al., 2015) should not be taken as a validation of the simulations’ adequacy to reproduce meaningful functional states. For instance, the coexistence of sparse firing and spontaneous emergence of propagating waves in the virtual brain has been seen by BBP as an encouraging proof of concept since they have been observed in the biological brain *in vivo* (Davis et al., 2020). However, similar spontaneous self-organizing processes have been described previously using large-scale modeling irrespectively of the grain chosen in the biological realism of the virtual brain circuitry and components (Izikevitch and Edelman, 2008; Davis et al., 2020; Jirsa, 2021). Despite the enormous work produced to create the most-ever-detailed simulation of a piece of sensory neocortex (Markram et al., 2015), the claim that the emerging ongoing activity in the *in silico* brain is equivalent to conscious brain activity is likely to push the digital predictions one bridge too far. It is fair to recognize that some key neuropsychologists and computational neuroscientists are convinced that *in silico* machines could one day become “conscious” (Dehaene et al., 2017), but the dominant arguments are presently based on mesoscopic brain imaging data (the read-out, and not the causal neural mechanisms) and use simplified modeling of thalamocortical and corticocortical dynamics (which do not require the detailed simulation of a full brain), disconnected from the cellular and subcellular levels (apart from the gating role of global neuromodulation).

Referring to the Turing-test used in artificial intelligence to answer the question: “can machines think?” (Turing, 1950), Koch and Buice (2015) proposed an imitation game to decide, on the basis of recordings, which are “biological” and which are *in silico*. Their clever review of BBP findings shows that the multiscale details of the cortical circuit optimized by BBP are probably irrelevant to some of the key predictions of the *in silico* simulations. Certain of the critical parameters controlling the excitability of the *in silico* network come from much simpler empirical models of synaptic neurotransmitter release and neuromodulation, and are not the emerging *de novo* consequence of the detailed network dynamics. The strategy recommended by Koch and Buice, to “add a mechanism if its impact on a specific set of measurable can be assessed by a trained observer,” is in fact very close to the Lego principle often used in computational neuroscience and neuroinformatics to complexify a first-order, simpler, computing architecture. The parametric sensitivity issue is particularly difficult to disentangle here, since the complex optimization process engineered by Markram and colleagues often deal with linked variables. The complexity issue of the model fitting does not stop there. How deep toward microscopic levels do you need to go to fit close to the biology: “It is not clear whether there is any “ultimate” level of reality (reductionism) where simulations abruptly bottom out” (Koch and Buice, 2015). Classical computational neuroscience studies have already shown that spike pattern reading is insufficient in comparing the brain and its *in silico* artifact. Voltage-clamp studies in vivo have demonstrated that, although the spike output may be the same, there are multiple ways to obtain the same spiking output through the nonlinear interaction between excitatory and inhibitory conductances (Frégnac and Bathellier, 2015). In particular, active “silent” states, such as produced by “silent” shunting inhibition, outsmart the imitation game if the chosen level of description does not allow to differentiate these from a passive resting state. One may conclude that optimization of parametric constraints is needed simultaneously at different levels of integration, but we are forced is to recognize that, at the present time, multiscale modeling is still at its infancy (Goldman et al., 2020).

## The 10-Year Prophecy

Ten years, the time needed to explore a myth? This slightly mystical fixed duration creates a strong feeling of urgency, remarkably similar to Ray Kurzweil’s announcement of a coming “singularity” (Kurzweil, 2005), when AI power, according to the prophecy, will surpass all human intelligence combined. Independently of the seriousness of this claim, the 10-year frontier also predicts the end of the project, hence establishing its totality in the global perspective and awareness of the public and the researchers involved. It also serves to convince funding agencies that the outcome will be reached in their life-time.

The fact that the delivery date of the final product of the project has been drifting further and further away, is not new in “Blue Sky” research (Bush, 1945). This often corresponds to the fact that the goal is more difficult to fulfill than initially thought. In the field of artificial intelligence, John McCarthy, Marvin Minsky, Nathaniel Rochester, and Claude Shannon, in the early 1950s, thought that artificial intelligence would succeed rapidly in simulating human intelligence: their initial bet was that it would take a two-month 10-man effort during a summer project to make significant advancement (McCarthy et al., 1955). Applying a fixed horizon to basic research also opens a circular argument. For instance, the Allen Institute was keen on defining sequences of 10-year phases in brain initiatives, allowing a change of focus once the first atlases (reachable deliverables) were on-line and the possibility to adapt scientific strategy at each new step to current developments.

On the one hand, a fixed horizon, too short to fulfill the ambition of the project, provides an easy getaway for the project initiators, if it turns out that there was not enough funding or if an effective synergy among participants is lacking. On the other hand, it is also a risk minimizing strategy, since one may have reasonable hope that technological breakthroughs will happen during the initial project time-span, calling for possible changes in the initial deliverable roadmap. For instance, the physical substrate of the micro-electronic chips, specifically used by the HBP neuromorphic platforms, may become technologically “dated” by the end of the game. A drawback, already experienced during pioneer FET project (Daisy, Facets, and BrainScales), is that the validation of this revolutionary form of computing (fast analog, asynchronous, parallel and distributed) requires enormous time and efforts to go further than the proof of concept (for review, see Furber, 2016; Ravindran, 2019). In parallel, it is likely that computing with Von Neumann architecture will adapt at a faster pace to the increase of electronic components performance, miniaturization and parallelism, predicted by Moore’s law. Consequently, traditional high-performance computing may still improve faster than what HBP can produce in terms of neuromorphic calculus power. This example shows the difficulty of designing and validating novel computing strategies directly inspired by the functioning of the brain during the 10 years of the project. It also explains why hybrid solutions (neuromorphic: BrainScales, vonNeumann-like: SpiNNaker) have been engineered in parallel during the entire duration of HBP to adapt to the ever-changing context of microelectronics.

Another secondary effect concerns the financier’s view point, since institutions may become aware, at some point in the 10-year cycle, that so much financial investment has been engaged that there is no longer a path of return. Consequently, once granted, whatever view point (the scientist’s or the funding agency’s), this type of megaproject is doomed to succeed, even if the final outcome is far from what was initially hoped. This view was shared by some of the EC-FET officers overseeing HBP. This suggests that at the end of the project, the fulfillment of the original promise may become irrelevant. In the worst case, “you can at least conclude that all you put into the model was not enough” (Markram, 2020a,b,c). What will count in the end will be (1) the tangible technological developments, some of them not even envisioned at the start of the venture, (2) the mixing of interdisciplinary communities who now have learned to work together, and (3) the future use, yet to be fully decided for in HBP, of the state-of-the-art infrastructures and archive access.

## The Flagship Way to “Singularity”?

Knowing what we know now, how can we summarize the essence of flagships in brain sciences? What do the associated tales tell us, that will help to sell science (Koksma, 2020)? Should flagships be a “Blue Sky” attempt to understand the brain? Should this research be led by theory, or solely motivated by a dream vision? Has the vision become simply a mediatized tool to encourage innovation and incentives to change scientific conduct, to better industrialize and merchandize the brain? Or is there something of a dual strategy in-between?

The promise-driven flagship, advocated so effectively by Henry Markram, calls for a new form of societo-scientific culture (Felt and Wyne, 2007; Jones, 2008; Panese, 2015; Ferry, 2016; Ganascia, 2017); that did not exist previously in brain sciences. The “economy of promises” revolves around a scientific or industrial process (or even a theoretical law) whose justification is not based just on scientific/technological arguments but on the promises themselves, as if their realization were guaranteed. In the IT world, it applies to Moore’s law, whose myth is perpetuated because of the commercial ambition of the designers of computing chips controlling at will the performance increase rate, hence the power-law of progress (Loeve, 2015; Ganascia, 2017). This new way of over-selling scientific targets, deeply aligned with what modern society expects from mega-sciences in the broad sense (big investment, big return), has been observed on several occasions in different scientific sub-fields of biology, such as nanotechnology, stem cells or synthetic biology, before invading the field of brain sciences and neuromarketing (Frégnac, 2017).

In the initial lobbying sessions organized by the EC in 2011 to evaluate the potential of large-scale research strategies, the flagship concept was often compared with three examples, taken from different fields:

### 

#### 

##### Astronautics (and Automobile Industry)

The race to the moon: the brain indeed is often presented as a world of uncharted territories, and the comparison with the “race to the moon,” or even “exploring the cosmos” in the words of Henry Markram, is tempting for Brain explorers. This “moon” banner was transformed into a “race to the Brain” by the Obama administration when passing funding for the BRAIN initiative (Alivisatos et al., 2013; who kept the BAM acronym of their President in the initial call’s title). The analogy with the motivation and the make-up of the full enterprise is however very weak, since the main motivation in reaching other planets with a habitable rocket was guided mostly by clear geopolitical ambitions and technological supremacy. The validation of the moon project was also well formulated: the safe return of astronauts! One may still wonder what could constitute an objective measure to validate BBP and HBP. The greater the knowledge we gather about the brain, the further the brain-moon/earth distance seems. The definition of a tangible return value, showing why the flagship (and not multiple interdisciplinary projects of smaller dimensions) was necessary, is still missing (Mainen et al., 2016).

##### Genetics: The Human Genome Sequencing Project

In contrast to BBP and HBP, this project was grounded from the start on the feasibility of a technological promise, although the expectations were high in the Health domain, specifically for the genetic dissection of Brain diseases and the causes of mental illness. It was the industrialization of technological methods and their application that led to the recognized success, and not the “Blue Sky” ambition, which was recognized later as naïvely formulated, considering the complexity of epigenetic and environmental factors (Collins et al., 2003; but see Chi, 2016; Roberts, 1990). On the one hand, this type of exhaustive dissection applied to terabytes of ultrafine serial section electron microscopy data may work successfully for paucineuronal networks and simple neural ensembles in invertebrates, even for full brains, as attempted (and succeeded) by Janelia Farm for the fruit fly (FlyEM Team; Scheffer et al., 2020). However, on the other hand, in the case of Human brain studies, it is unlikely that a brute force approach of the same type, as practiced by BBP, would ensure significant progress in understanding the fundamental nature of mind processes.

##### Physics: the CERN platform

The inspiration here comes from particle physics, a field which, in contrast to brain sciences, has an impressive record in large-scale projects. Theorists are actively involved in the design of collective infrastructures and mega-equipment shared by the entire experimental community, such as, for example, the large hadron collider (LHC), the CERN particle detectors (Atlas), and laser interferometer gravitational waves (LIGO).

The comparison made between CERN and a “mind observatory,” “mindscope,” or “neuroscope,” used rhetorically by the Allen Institute or BBP/HBP, is, to my view, misleading. The mega-science infrastructures in physics derive immediate benefit from “unique” shared instruments, designed in collaboration to collect new experimental data and test explicit hypotheses in the light of a fundamental global theory. Theorists are involved before the data-collection stage, and not after, as is the case in data-driven brain science initiatives, and participate in elaborating experimental protocols designed to validate or invalidate the predictions of their theories. The construction of a massive architecture of databases collected without a theoretical framework could turn into a waste of energy, time and money (Frégnac and Laurent, 2014; Mainen et al., 2016).

Still, the prevailing message coming from both BBP and HBP is nowadays that the enterprise will succeed in producing a “viewing neuroscope” IT platform built largely on preexisting data. In his *Science* interview in 2011, Henry Markram already toned down the ambition of the initial project. “What we have been doing, contrary to what most people think we’ve been doing, is not just building a model. We’re building a facility to build brain models. It’s much more about a strategy for data integration.” “What is difficult to get across to the public is that the end result of what we build is going to be far more boring than they would hope. It is going to be like a massive telescope or an MRI machine sitting in a hospital, and scientists will get together to write a proposal and they’ll book half a day on the machine to run a simulation to test a particular hypothesis (Markram, 2011).” Progress is thus expected mostly away from the experimental bench, and gained from the alliance of deep learning, neuroinformatics and neuromorphic computation, promised to be significant enough to sustain virtual medicine applications (Markram, 2013; Sanz-Leon et al., 2013).

A secondary effect of what many consider as over-selling arguments is that similar considerations are now used by governmental institutions in Europe and the United States to suggest that enough experimental data may be already available on the laboratory shelves, constituting a pile of “siloed” dormant sources that need to be curated. Will this analysis prevail in the long run and spell the end of animal experimentation, in particular in non-human primates, a trend already present in European research policy? This may raise considerably the stakes concerning the biological relevance of the digital brain and the achievements of *in silico* simulation.

## Back to Earth

Ten years have passed. The “visionary” dimension of the objectives of the original promise is long gone. The “Blue Sky” and “interdisciplinarity” dimensions, which were the key features of the ICT-FET vision of the EC, are under test and possibly in jeopardy, since most of the supranational funding for Brain Sciences in Europe has been swallowed by the HBP flagship and will not be renewed as such. A problematic concern remains the over-promising of the flagship objectives, resulting from the extreme level of competition generated by the announced size of the funding (projects each requesting up to a billion Euros). Irrespective of what happens in the end, the story of Science is pulsed and propelled by strong beliefs. In the closing statements of *In Silico*, Henry Markram says that this journey: “will lead to success, with himself or others. It will be more significant than landing on the moon. It improves every year and perfection is at the end of the path.”

The revised end-point for BBP is now officially in 2050, a rounder number, just after the date at which Kurzweil predicted “singularity” will be reached. Is this a sign that our brain will be one day fully digitized, assuming that Kurzweil and Elon Musk win their bets? Or is it just a convenient way to avoid answering the question, the one with which we started: how blue-sky should research be, if we really want to better understand the Brain?

## In Insert: The *In Silico* A Propos

*In Silico* (Hutton, 2020) tells the story of the progressive disillusion of a young film-maker in his twenties who cherished the hope of making a longitudinal narrative of a 10-year futuristic project led by a charismatic leader. The promise that Henry Markram made in his illustrious 2009 TED talk, to build a working digital version of the human brain in the next 10 years, struck Noah Hutton with a vision: he wanted, one way or another, to be part of the journey, and, to do so, would film the epic, from start to finish, from an insider’s immersive perspective. As year followed year, his own belief in the feasibility of the initial claim gradually evolved and eroded, challenged by the waves of criticism he recorded in multiple interviews with highly educated minds, and by the difficulties that the Blue Brain researchers (mostly EPFL scientists and engineers) encountered step after step in their tasks. The film ends after 10 years, at a stage where the head of the virtual brain simulation and the head of neurorobotic implementations, both key BBP members, are leaving the Ark and where deadlines dissolve, drifting into a reshapable future.

In terms of movie-making and storytelling, Noah Hutton provides an interesting, but limited, one-sided single observer’s view. The complexity of the story calls for a more comprehensive multi-facetted collection of narratives, in the style of *Rashomon or The Gates of Hell* (1950, directed by Akira Kurosawa), where the subjective views of the same event are replayed in succession through the eyes of each of the main active protagonists, and not only of a passive witness. In terms of the history of scientific history, *In Silico* does not give a fair account of journeys traveled by the BBP or HBP journey, because it is incomplete. By design, the documentary is centered on only two personalities, the project leader and the cineaste observer. It bears little on the scientific or technological achievements themselves.

However, the film does enlighten another dimension, mostly societal, and gives clues to why there has been such a profound fracture between the neuroscience community and the BBP. This chasm is almost physical, defining who is “in” and who is “out.” Clearly, the thick bank-like walls of the EPFL define a physical limit, fencing the borders of the Village Vaudois (as attested by the BBP participants and the Asterix t-shirt metaphor in the film). Noah Hutton recognizes that “seeing things done in the Blue room made me believe more.” The graphic engineer, entranced in a smooth Tai-chi-like dance to animate the viewing perspective, brings to mind Michael Douglas immersing himself in the data files libraries in the movie *Disclosure*. The intention is to lead the viewer in the brain, to the point where the virtual brain “becomes your home.” In short, the “blue room” is the metaphor for who is “in” and “sees the brain,” and who remains “out” of the glass building of EPFL and will not reach the truth!

To some viewers, Noah Hutton’s enterprise may seem lacking ambition and objectivity: it gives a factual account of a scientific project, seen through a societal peephole focusing on the characters who are driving it. In this respect, its reception may be mixed, as that of Susan Allport’s earlier longitudinal study of the search for cellular correlates of learning and classical conditioning (Allport, 2001), dealing with high profile egos specialized in the invertebrate world [Daniel Alkon and (at that time future) Nobel Prize winner, Eric Kandel]. But, interestingly enough, Noah Hutton’s film raises deep epistemological questions: where does the driving force of flagship projects, such as the BBP or the HBP, come from: a scientific revolutionary idea, a paradigm shift, or the intuition of a charismatic leader? a winning industrial strategy or the search for a speculative bubble burst? Why do such projects set out to last 10 years? What if they do not succeed, or not yet?

## Synthesis

Reviewing Editor: Christophe Bernard, INSERM & Institut de Neurosciences des Systèmes Decisions are customarily a result of the Reviewing Editor and the peer reviewers coming together and discussing their recommendations until a consensus is reached. When revisions are invited, a fact-based synthesis statement explaining their decision and outlining what is needed to prepare a revision will be listed below. The following reviewer(s) agreed to reveal their identity: NONE.

N/A
